# Practical Pathology

**Published:** 1885-03

**Authors:** 


*Practical Pathology.* By G. SIMS WOODHEAD, M.D. Second Edition. Pp. 534. Edinburgh: YOUNG J. PENTLAND. 1885.

The very high opinion as to the merits of this work which we have already expressed has been more than confirmed by the rapid call for a new edition. Careful revision, the rewriting of some parts, and the addition of much new matter, have still further added to the excellence of the work.

We would again call attention to the very superior manner in which the publisher has performed his part of the work. We have seen no medical handbook produced in England with so much combined excellence of printing, paper, binding, and engraving. It is positively a pleasure to handle the book.

